# Low oxygen sensing and balancing in plant seeds: a role for nitric oxide

**DOI:** 10.1111/j.1469-8137.2007.02226.x

**Published:** 2007-12

**Authors:** Ljudmilla Borisjuk, David Macherel, Abdelilah Benamar, Ulrich Wobus, Hardy Rolletschek

**Affiliations:** 1 Institut für Pflanzengenetik und Kulturpflanzenforschung (IPK)Corrensstr. 3, 06466 Gatersleben, Germany; 2 UMR Physiologie Moléculaire des Semences (Université d'Angers/INH/INRA)ARES, 16 bd Lavoisier, 49045 Angers cedex 01, France

**Keywords:** ATP availability, crops, hypoxia, membrane inlet mass spectrometry (MIMS), oxygen sensor, microsensor, seed photosynthesis, storage metabolism

## Abstract

- Storage product accumulation in seeds of major crop species is limited by their low internal oxygen concentration. Adjustment of energy and storage metabolism to oxygen deficiency (hypoxia) in seeds is highly relevant for agriculture and biotechnology. However, the mechanisms of low-oxygen sensing and balancing remain a mystery.
- Here, it is shown that normal hypoxia in seeds of soybean (*Glycine max*) and pea (*Pisum sativum*) triggers a nitrite-dependent increase in endogenous nitric oxide (NO) concentrations. NO, in turn, reduces the oxygen consumption of seeds, generating a localized decrease in both ATP availability and biosynthetic activity. Increasing oxygen availability reduces endogenous NO concentrations, thereby abolishing mitochondrial and metabolic inhibition.
- This auto-regulatory and reversible oxygen balancing, via NO, avoids seed anoxia and suggests a key role for NO in regulating storage activity. This hypothesis is reinforced by changes in energy status (ATP:ADP ratio), steady-state metabolite concentrations and biosynthetic fluxes under NO treatment.
- The proposed mechanism of low-oxygen sensing and balancing in plants offers the prospect of a new field of study in crop biotechnology.

*New Phytologist* (2007) **176**: 813–823

## Introduction

Plant roots and other submerged organs are frequently challenged with limited oxygen supply (Armstrong *et al*., 1994; Crawford & Brändle1996; Drew1997). Restricted capabilities for oxygen diffusion and high rates of cellular metabolism may cause hypoxia even in aerial organs. This is especially true for developing plant seeds, including the major crop species maize (*Zea mays*), soybean (*Glycine max*), oilseed rape (*Brassica napus*), barley (*Hordeum vulgare*), wheat (*Triticum aestivum*), pea (*Pisum sativum*) and sunflower (*Helianthus annuus*). Their cellular environment is characteristically low in oxygen (Table 1). Despite clear species differences in morphology (i.e. gas exchange capability), photosynthetic ability, respiration, and so on, seeds tune their steady-state oxygen concentration to a ‘basic’ concentration of approx. 2–10 µm (Table 1). This oxygen concentration might represent a compromise between avoiding the risk of severe anoxia and supporting maximum metabolic activity. How this concentration is balanced remains unclear.

**Table 1:** Overview of internal oxygen concentrations within crop seeds

Species	O_2_ concentration (µm)	Remarks	Reference
Broad bean* (*Vicia faba*)	9	O_2_ concentration depends on developmental stage and increases under light	Rolletschek *et al*. (2002)
Pea* (*Pisum sativum*)	2	Minimum O_2_ at early developmental stages; O_2_ increases under light	Rolletschek *et al*. (2003)
Soybean* (*Glycine max*)	2	Transient increase in O_2_ under light is followed by a compensatory rise in respiratory activity	Rolletschek *et al*. (2005a)
Rapeseed* (*Brassica napus*)	10	No data on light/stage dependence	Vigeolas *et al*. (2003)
Barley† (*Hordeum vulgare*)	3	Deep O_2_ gradients under light conditions; O_2_ concentration within endosperm decreases at main storage stage and increases slightly under light	Rolletschek *et al*. (2004)
Wheat† (*Triticum aestivum*)	25	Measurements during light phase; no data on developmental changes	VanDongen *et al*. (2004)
Maize‡ (*Zea mays*)	4	O_2_ concentration depends on developmental stage but no light effects; minimum concentrations in starchy endosperm but higher in embryo	Rolletschek *et al*. (2005b)
Sunflower‡ (*Helianthus annuus*)	3	O_2_ concentration depends on developmental stage but no light effects	Rolletschek *et al*. (2007)

The mean O_2_ concentration was measured at ambient air under dark conditions (except for wheat) using fibre-optic microsensors.

* The endogenous oxygen concentration depends strongly on light supply.

† The green pericarp surrounds the nongreen starchy endosperm, and supplies significant amounts of photosynthetically released oxygen towards the interior.

‡ Nongreen seeds are fully dependent on diffusive oxygen uptake and thus lack any light dependence of their oxygen status.

In addition to frequent hypoxia, seeds must also accommodate their respiratory/metabolic activity to ambient light and other environmental inputs. A striking example is offered by the highly photosynthetically active seed of oilseed rape. Insertion of O_2_-sensitive microsensors into seeds reveals that light/dark switches generate large, reversible and very rapid fluctuations in internal oxygen concentrations, from strong hypoxia (< 1 µm in dark conditions) to hyperoxia (> 700 µm; see Supplementary Material Fig. S1). In seeds of soybean, the endogenous oxygen concentration rises upon illumination, but falls to resting values after some period of light adaptation. Thus, rising O_2_ supply is apparently balanced by increasing O_2_ consumption, that is, mitochondrial respiration (Rolletschek *et al*., 2005a). How such adaptive responses are regulated at the molecular level is unknown.

In the absence of oxygen, the mitochondrial ATP supply will be inhibited because oxygen is the terminal electron acceptor in the respiratory chain. Hence, it is not surprising that the imposition of hypoxia leads to a rapid decrease in both the availability of ATP and biosynthetic fluxes (Geigenberger2003; Greenway & Gibbs2003; Rolletschek *et al*., 2003), as well as affecting gene expression more generally (Chang *et al*., 2000; Klok *et al*., 2002; Liu *et al*., 2005). However, the inhibition of mitochondrial respiration occurs at oxygen concentrations much higher than the Michaelis constant (*K*_m_) value of cytochrome C oxidase. Both the molecular mechanisms underlying these reactions and the molecular means of oxygen sensing/signalling are, in contrast to the case in animals, largely not understood in plants. There is some evidence for *indirect* oxygen sensing based on changes in cellular homeostasis (for a review, see Bailey-Serres & Chang2005). However, *direct* oxygen sensors capable of detecting/reacting with oxygen and triggering a signalling cascade have not been found to date, despite tremendous research efforts. This leads to the speculation that such sensors either do not exist in plants or are of a different nature (e.g. small, inorganic molecules instead of complex proteins).

Nitric oxide (NO) is a gaseous free radical, sensitive to the presence of molecular oxygen. In mammals, it modulates blood pressure, oxygen consumption and other physiological functions (Stamler & Meissner2001; Moncada & Erusalimsky2002) via complex signalling cascades (Wendehenne *et al*., 2001). In plants, NO has been implicated in various aspects of physiology, including plant–pathogen interactions (Delledone *et al*., 1998) and mitochondrial function (Yamasaki *et al*., 2001; Zottini *et al*., 2002). However, there are no reports relating NO to energy and storage metabolism in seeds. When hypoxia was imposed on roots of alfalfa (*Medicago sativa*), NO production was increased (Dordas *et al*., 2003). Hypoxia also induces the accumulation of nonsymbiotic class 1 haemoglobins, which have been shown to possess NO deoxygenase activity (Perazzolli *et al*., 2004). Recent evidence suggest that nonsymbiotic haemoglobins use NADPH to convert NO to nitrate. Associated with the oxidation of NADH and the haemoglobin/NO cycle is the maintenance of the redox and energy status of the cell under hypoxic conditions (Igamberdiev *et al*., 2005).

On the basis of the chemical nature of NO (its sensitivity to molecular oxygen) and its effects on mitochondrial functionality (inhibition of cytochrome C oxidase), we propose a key role of NO in immediate sensing and balancing of the oxygen status in plant seeds. Here, we used microsensor- and mass spectrometry-based quantifications of NO and O_2_ to reveal the relationship of hypoxia (O_2_ availability) and endogenous NO. NO mediates reversible oxygen balancing via its effect on respiratory activity, and enables the seed to avoid endogenous anoxia. Thereby, NO also controls energy availability for storage product synthesis, a central process with respect to agricultural food production.

## Materials and Methods

### Plant growth

Plants of soybean (*Glycine max* (L.) Merr.), pea (*Pisum sativum* L.) and oilseed rape (*Brassica napus* L.) were cultivated in a glasshouse under natural light supplemented with lamps to provide a 16:8-h photoperiod and an approximate light intensity of 800 µmol photons m^−2^ s^−1^. Temperature was controlled between 25 and 30°C. Seeds undergoing their main storage phase were used for this study.

### Measurement of nitric oxide and oxygen concentrations in seeds

The concentration of endogenous NO in the seeds was measured using an NO electrode (100-µm tips; ISONOPF 100; WPI, Berlin, Germany), connected to an Apollo 4000 system (WPI). The electrode was calibrated using freshly prepared NO-saturated water (NO gas from Messer-Griesheim, Berlin, Germany). The intact seed was carefully moved into a fixed position. Then, the electrode was inserted into the seed using a micromanipulator. Note that the NO sensor enables one to determine concentration changes but not absolute NO concentrations within the seed tissue, because moving the sensor from the calibration solution to the seed changes the absolute sensor signal (but not the relative changes). The oxygen concentration inside seeds was measured using oxygen-sensitive microsensors (30-µm tips; Presens, Neuburg, Germany) attached to a micromanipulator (for details, see Rolletschek *et al*., 2003). To alter endogenous oxygen concentrations, intact seeds were aerated with either gaseous nitrogen or oxygen (Messer-Griesheim, Magdeburg, Germany). In the case of rapeseed, the light supply during measurements was varied from 400 to < 2 µmol photons m^−2^ s^−1^.

### Effects of nitric oxide on endogenous oxygen concentrations and energy status

Intact seeds of pea and soybean were isolated. Exogenous NO was supplied to the seed by injecting trace amounts (0.1–2 µl) of NO-saturated or distilled water (as a control) with a syringe into the seed. At specific time intervals thereafter, the seeds were frozen in liquid nitrogen and stored at –80°C until required for analysis. In some cases, the oxygen-sensitive microsensor was inserted into the seed beforehand, and the effect of NO injection on the internal oxygen concentrations was monitored.

### Determination of nitric oxide emissions by membrane inlet mass spectrometry

The NO emission of isolated embryos was monitored using membrane inlet mass spectrometry (MIMS; Conrath *et al.*, 2004). The plant material was incubated in closed chambers with 15 ml of buffer containing 80 mm sucrose, 30 mm KH_2_PO_4_ and 20 mm 2-(N-morpholino)ethanesulfonic acid (MES), pH 6.5, in either light or darkness at 20°C. The method allows the simultaneous determination of NO and oxygen concentrations in the incubation solution. From the decrease of oxygen within the buffer solution over time, the (mainly respiratory) seed oxygen uptake was calculated. The reversibility of NO-induced reductions in seed oxygen uptake was assessed using an NO scavenger (2-phenyl-4,4,5,5-tetramethylimidazoline-1-oxyl 3-oxide; PTIO). Chemicals were purchased from Sigma (Seelze, Germany) except for ^15^N-nitrite (Rotem GmbH, Leipzig, Germany). In some cases, buffers were deoxygenated by bubbling with gaseous nitrogen for 1 h.

### Mitochondrial isolation and oxygraphic measurements

Intact mitochondria were isolated from dry mature pea seeds after 22 h of imbibition and purified using a combination of step and self-generated gradients of Percoll (Amersham Biosciences Europe, Saclay, France) as described previously (Benamar *et al*., 2003). The oxygen consumption of mitochondria was monitored with an oxygen electrode system (Oxytherm; Hansatech, King's Lynn, UK). The electrode medium contained 0.6 m mannitol, 20 mm MOPS (pH 7.5), 10 mm KH_2_PO_4_, 10 mm KCl, 5 mm MgCl_2_, and 0.1% (weight/volume (w/v)) bovine serum albumin (BSA). For oxidation of malate-glutamate (7.5 mm each), the medium was supplemented with 1 mm NAD, 0.3 mm thiamine pyrophosphate, 50 µm coenzyme A, 1 mm pyruvate and 5 mm DTT. The protein concentration was determined by a modified Lowry assay (RC DC Protein Assay; BioRad, Hercules, CA, USA) using BSA as a standard.

### ATP imaging

Local ATP concentrations were analysed by quantitative bioluminescence imaging as described previously (Borisjuk *et al*., 2003).

### *In vitro* culture

Isolated embryos of pea and soybean were incubated in 20 ml of buffer solution containing 100 mm sucrose, 25 mm glutamine, 25 mm asparagine, 15 mm KH_2_PO_4_ and 10 mm MES buffer (pH 6.5). Using a minute-injection system (11Plus; Harvard Apparatus, Holliston, MA, USA), NO-saturated water was added to the buffer at a constant flow rate of 2 µl min^−1^ giving a stable NO concentration of *c*. 1.5 µm. After 1 h of incubation under light (approx. 200 µmol photons m^−2^ s^−1^), the embryos were collected for metabolite analysis and frozen in liquid N_2_.

### Metabolite analysis

Plant material was homogenized with a pestle and mortar and extracted with a chloroform/methanol mixture (Soga *et al*., 2002). Dissolved sugars were measured photometrically (Rolletschek *et al*., 2005b), and adenine nucleotides were detected fluorescently using high-performance liquid chromatography (HPLC) after derivatization (Rolletschek *et al*., 2005a). Glycolytic intermediates and organic acids were measured by ion chromatography coupled to mass spectrometry (Rolletschek *et al*., 2005b).

### Stable isotope labelling

For stable isotope labelling, intact pea embryos were incubated as above in a buffer containing additional 10 mm^13^C-sucrose (Omicron Biochemicals, South Benol, IN, USA) and 10 mm^15^N-glutamine (CDN Isotopes, Quebec, Canada). After 5 h of incubation, embryos were frozen in liquid N_2_. Subsequently, the samples were homogenized and extracted twice with 1 ml of 60% (v/v) ethanol and once with 1 ml of H_2_O. To dissolve protein, the insoluble material was incubated for 24 h at 30°C in 1.5 ml of 50 mm Tris-HCl (pH 7.4) containing 0.08% (w/v) pronase (Sigma). After centrifugation (5 min at 14 000 ***g***), the supernatant was collected and the pellet washed twice in 1 ml of H_2_O. To hydrolyse starch, the remaining insoluble material was incubated with 14 U of amyloglucosidase in 1 ml of 50 mm sodium acetate (pH 4.8) for 24 h at 55°C. After centrifugation (10 min at 14 000 ***g***), the supernatant was collected. A 500-µl aliquot of either the protein or starch-containing supernatants was transferred to stannous foil, dried and subsequently analysed for its content of isotope pairs (^12/13^C and ^14/15^N) using elemental analysis (Vario EL3; Elementaranalysesysteme, Hanau, Germany) coupled to isotope ratio mass spectrometry (ESD 100; IPI, Bremen, Germany).

### RNA isolation and hybridization

RNA from pea embryos was isolated and northern hybridizations were performed using standard procedures and cDNA fragments as described previously (Weber *et al*., 1995).

## Results and Discussion

### Seeds respond to low oxygen with elevated nitric oxide concentrations

**Fig. 1:**
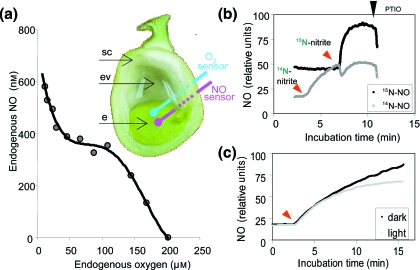
Dynamics of endogenous nitric oxide (NO) concentration and NO release measured in pea (*Pisum sativum*) seeds using microsensors (a) and mass spectrometry (b, c). (a) The diagram illustrates the experimental set-up for microsensor measurements: after insertion of the sensors, the intact seed was aerated with either nitrogen or oxygen, and variation in the endogenous concentrations of NO and oxygen was monitored. The graph shows the endogenous NO concentration in response to changes in the internal oxygen concentration. (b) Release of either ^14^N- or ^15^N-labelled NO from *in vitro* incubated pea embryos after addition (red arrows) of 750 nmol ^14^/^15^N-labelled nitrite. The effect of the addition of 2-phenyl-4,4,5,5-tetramethylimidazoline-1-oxyl 3-oxide (PTIO; 5 µmol) demonstrates the NO specificity of the signal. For details, see the Materials and Methods. (c) NO release from pea embryos incubated *in vitro* under either light or dark conditions. The red arrow indicates the addition of 750 nmol nitrite. e, embryo; ev, endospermal vacuole; sc, seed coat.

To evaluate whether changing oxygen concentrations affect the internal concentration of NO in seeds, we simultaneously monitored changes in the steady-state concentrations of NO and oxygen. For this purpose, two microsensors, one sensitive to NO and the other to oxygen, were inserted into intact pea seeds maintained in the dark (scheme shown in Fig. 1a). To alter internal oxygen concentrations (mimicking photosynthetic oxygen release), the seeds were aerated with either gaseous oxygen or nitrogen. Increases in the endogenous oxygen concentration (equivalent to photosynthesis in the light) decreased endogenous NO to below the level of detection, while lowering the endogenous oxygen concentration strongly increased the concentration of NO (Fig. 1a). Importantly, these changes occurred within minutes of the treatment being applied. Soybean seeds behaved in a very similar way (data not shown). The rise in endogenous NO at low oxygen availability may be achieved through the *de novo* synthesis of NO and/or as a result of increasing NO stability (less quenching at low oxygen).

Because of their central importance for our hypothesis, the microsensor measurements of NO were confirmed using an alternative approach: the NO emission of isolated pea embryos was measured by MIMS (Conrath *et al.*, 2004). NO evolution by *in vitro* incubated embryos was only detectable after the addition of nitrite. Importantly, the NO release of embryos was greatly stimulated under anoxic vs nonanoxic conditions (Supplementary Material Fig. S2). This supports the notion of an O_2_ dependence of endogenous NO concentrations, and is consistent with the behaviour of leaves (Planchet *et al*., 2005) and roots (Dordas *et al*., 2003).

To determine the *in vivo* substrate for NO synthesis in seeds, we also used the MIMS technique. The release of NO from *in vitro* incubated pea and soybean embryos was measured following the addition of various substrates. The MIMS assay allows simultaneous monitoring of both ^14^N- and ^15^N-labelled NO. Notably, before addition of nitrite, the steady-state signal for NO was approx. 24 and 50 (relative units) for ^14^N- and ^15^N-NO, respectively (Fig. 1b). The higher signal for ^15^N-NO was caused by the distinct mass trace sensitivity of the mass spectrometer. Following the addition of either ^14^N- or ^15^N-labelled nitrite, NO release by pea embryos was strongly induced. Depending on which nitrite had been added, the released NO contained either ^14^N or ^15^N, clearly indicating that nitrite is directly used as a substrate for NO synthesis in seeds. Similar data were derived from soybean embryos (not shown). In contrast, the addition of neither arginine nor nitrate (alternative substrates for NO synthesis; see Wendehenne *et al*., 2001) had any detectable effect on NO release (data not shown). This might imply that NO synthesis in seeds is mediated via nitrite-utilizing enzymes, and is consistent with the fact that the concentration of nitrite was shown to increase under hypoxia (Botrel *et al*., 1996), providing a substrate for NO synthesis. It also corresponds to recent findings showing that plant mitochondria have the capacity to convert nitrite into NO under anaerobic conditions to drive ATP synthesis (Stoimenova *et al*., 2007). In the absence of nitrite, of course, other substrates might also play a role in NO synthesis *in vivo*.

As light strongly influences the concentration of endogenous oxygen in immature green seeds via photosynthesis (Supplementary Material Fig. S1, and Rolletschek *et al.*, 2003, 2005a), we studied the effect of light on NO emission. When pea embryos were incubated in the MIMS chamber, the addition of nitrite led to an instantaneous rise in NO emission, this increase being greater under nonlit than under lit conditions (Fig. 1c). We propose that the light dependence of NO emission is related to changes in the concentration of endogenous oxygen.

We conclude that the endogenous NO concentration in seeds responds instantly to oxygen availability.

### Nitric oxide diminishes the oxygen uptake of seeds

**Fig. 2:**
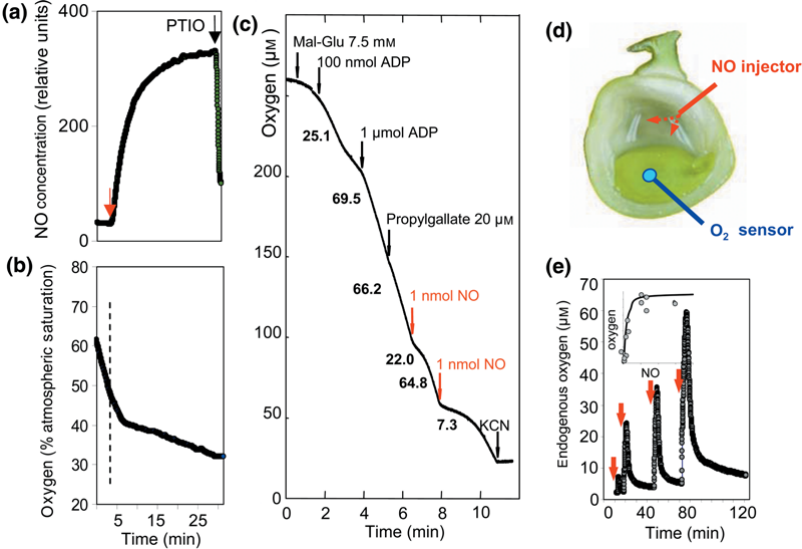
The effect of nitrite and nitric oxide (NO) on oxygen uptake, mitochondria and oxygen status of pea (*Pisum sativum*) seeds. (a, b) Pea seeds were incubated *in vitro* in the membrane inlet mass spectrometry (MIMS) chamber. After the injection of 750 nmol nitrite (red arrow), the NO concentration increased, indicating the NO release by the embryos. With some time delay, the oxygen uptake rate (estimated from the O_2_ decline over time) decreased. The dotted line in (b) indicates the time of nitrite addition. The addition of 2-phenyl-4,4,5,5-tetramethylimidazoline-1-oxyl 3-oxide (PTIO; 5 µmol) was used to demonstrate the NO specificity of the signal. (c) The graph shows oxidation of malate-glutamate (Mal-Glu) by intact mitochondria isolated from pea seeds; arrows indicate the addition of the various compounds, and numbers under the traces refer to the rates of oxygen consumption in nmol min^−1^ mg^−1^ protein. Note that the inhibitory effect of NO on cytochrome oxidase was reversible, but it was greater at lower oxygen concentrations (22.0 vs 7.3 nmol min^−1^ mg^−1^ protein). (d) The experimental set-up for the NO microinjection experiment: after insertion of the oxygen-sensitive microsensor, trace amounts of NO were injected into intact seeds, and endogenous oxygen concentration changes were monitored. (e) Changes in endogenous oxygen concentration in response to the injection of 20, 60, 100 and 300 pmol NO (as indicated by red arrows); the insert shows the dose–effect curve for multiple NO injections.

We next determined whether NO affects the seed oxygen balance. Nitrite was added to the nutrient buffer bathing the pea embryos, while the NO and oxygen concentrations in the buffer were monitored by MIMS. The addition of nitrite induced an immediate release of NO (Fig. 2a), and resulted in a decrease of *c*. 80% in oxygen uptake by the embryo (Fig. 2b), effects that became apparent after *c*. 1.5 min. Under control conditions (without added nitrite), the oxygen uptake of the embryo was steady (data not shown). Adding trace amounts of NO (rather than of the NO substrate nitrite) resulted in a similar decrease in embryo oxygen uptake (Supplementary Material Fig. S3a,b), while the addition of the NO scavenger PTIO abolished the NO inhibition in a nearly completely reversible fashion. The addition of 95, 190 or 570 nmol NO inhibited the oxygen consumption rate by, respectively, 70, 77 and 81% and was thus dosage dependent.

Total oxygen uptake is mainly determined by mitochondrial respiration, and NO is known to affect mitochondrial function, and in particular to reversibly inhibit the cytochrome pathway in the mitochondrion (Millar & Day1996; Yamasaki *et al*., 2001; Huang *et al*., 2002; Zottini *et al*., 2002). Intact mitochondria isolated from pea embryos were tested (Fig. 2c) to evaluate the NO action in seeds. The addition of propylgallate, an inhibitor of alternative respiration, had little effect on the electron transfer driven by malate-glutamate oxidation, indicating a low activity of the alternative respiratory pathway in seeds. However, the addition of NO produced a significant and reversible inhibition of mitochondrial respiration. Oxygen consumption rates declined from 66.2 to 22.0 nmol oxygen min^−1^ mg^−1^ protein, but recovered to resting values (64.8 nmol oxygen min^−1^ mg^−1^ protein) within 2 min. This effect of NO on seed respiration basically confirms earlier findings. However, the important finding is that the inhibitory effect of NO was dependent on the oxygen concentration, that is, it was highest at the lowest oxygen concentration (7.3 vs 22.0 nmol oxygen min^−1^ mg^−1^ protein; Fig. 2c). In a similar way, in mammalian cells, NO reduces the affinity (i.e. increases the *K*_m_) of cytochrome C oxidase for oxygen (Moncada & Erusalimsky2002). This modulation of *K*_m_ may in part explain the onset of respiratory restriction at relatively high oxygen tensions (Geigenberger2003). In summary, we propose firstly that released NO (the release having been induced by a low-oxygen environment) reversibly inhibits seed oxygen consumption, and secondly that the extent of this inhibition is regulated by the endogenous oxygen tension. Through this NO-driven, auto-regulatory mechanism, the oxygen demand of seeds can be dynamically adjusted according to the ambient oxygen concentration, thereby avoiding the descent into anoxia. The NO-mediated stimulation of respiration through the alternative pathway (Huang *et al*., 2002) is not likely to be of any great significance in seeds, given their low activity of alternative oxidase. Overall, the data suggest that endogenous NO diminishes oxygen uptake of seeds via its effects on mitochondrial respiration.

### An auto-regulatory mechanism for oxygen balancing in seeds

To test the assumption that oxygen availability in seeds is influenced by NO-mediated changes in oxygen consumption, short-term changes of endogenous oxygen in seeds in response to NO treatment were monitored. An oxygen-sensitive microsensor was inserted into the embryo of an intact pea seed, and trace amounts of NO were injected using a microsyringe into the endospermal vacuole surrounding the embryo (Fig. 2d). The oxygen concentration rose immediately after NO injection, but returned rapidly to close to its initial values (Fig. 2e). This effect was dosage dependent. The reversibility of this pattern is consistent with the quenching of NO by endogenous oxygen, and is in good agreement with measurements made on isolated mitochondria (Fig. 2c). It implies that increases in NO reduce oxygen consumption. Finally, the availability of oxygen is increased. Analogous conclusions have been drawn in mammalian cell studies using a bioluminescence assay (Hagen *et al*., 2003).

Under *in vivo* conditions, the NO-mediated repression of respiration is assumed not to increase, but rather to stabilize the tissue oxygen concentration at a low level. This corresponds to a compensatory balancing of the oxygen:NO ratio. We conclude that the seed uses the oxygen:NO ratio to reversibly adjust oxygen supply to oxygen demand. It enables the seed to tightly regulate cellular respiration. Importantly, this NO-dependent, auto-regulatory mechanism is driven by the oxygen tension itself.

### Nitric oxide regulates local ATP availability and biosynthetic activity of seeds

**Fig. 3:**
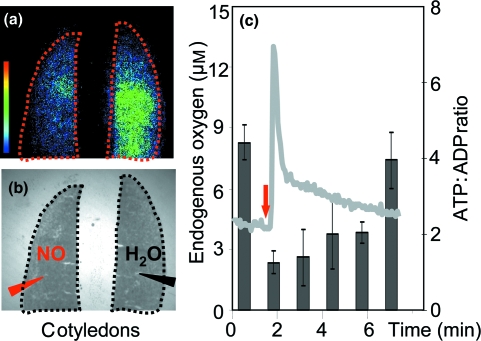
The *in vivo* effect of nitric oxide (NO) addition on the energy status of soybean (*Glycine max*) seeds. (a, b) Image of ATP distribution within the embryo (a). Analysis was done by quantitative bioluminescence using cryosections of embryo as shown in (b). The embryo was treated with either NO (microinjection of 1 nmol in 1 µl; red arrow in left cotyledon) or distilled water (microinjection of 1 µl; black arrow in right cotyledon); the colour scale represents the ATP concentration in relative units, from low (blue) to high (red). (c) A time course of the ATP/ADP-ratio (bars) and oxygen concentration (curve) measured before and after NO injection. The red arrow indicates the time of NO injection (1 nmol) into the intact seed. Embryos were sampled at distinct time intervalls. The oxygen concentration within the embryo was measured using microsensors in a parallel experiment. Note the reversibility of both energy and oxygen status. Bars are means ± SD.

To test whether the NO effect on mitochondria transmits to the energy status of seeds, we analysed the adenylate pools after NO treatment, both spatially with quantitative bioluminescence and temporally with HPLC. The left and right cotyledons of a soybean embryo were injected with, respectively, NO (approx. 1 nmol) and distilled water (as a control). The metabolism was quenched by freezing the embryos in liquid nitrogen. After cryosectioning of the embryo, tissue slices (shown in Fig. 3b) were processed using a quantitative bioluminescence assay (for details, see Borisjuk *et al*., 2003). It became obvious that the NO treatment induced drastic reductions in local steady-state ATP concentrations, compared with those in the control (Fig. 3a). The reduction in ATP was evident already after 15 s of incubation, indicating a very rapid NO-mediated regulation of ATP supply. This can be explained by the binding of NO to cytochrome C oxidase, which reduces electron transport and thus ATP synthesis in both plant (Yamasaki *et al*., 2001) and mammalian (Moncada & Erusalimsky2002) mitochondria. It remains to be said that *in vitro* results should be interpreted with some caution. Clearly, the concentrations and locations of injected NO are unlikely to be the same as those found *in vivo*.

The decrease in ATP concentrations was also reflected in the kinetics of the ATP:ADP ratio, which characterizes the energy status of the tissue (bars in Fig. 3c). Following the injection of 1 nmol NO, the resulting oxygen concentration curve is indicative of the extent of respiratory inhibition. In parallel with the rise in oxygen concentrations, the ATP:ADP ratio fell immediately after NO injection, but recovered to close to its initial values after 10 min. Within this time, the concentration of oxygen had also more or less reached its resting concentration, indicating the almost complete reversibility of NO action. We conclude that hypoxia-induced increases in endogenous NO concentration modulate the availability of ATP within the seed.

**Fig. 4:**
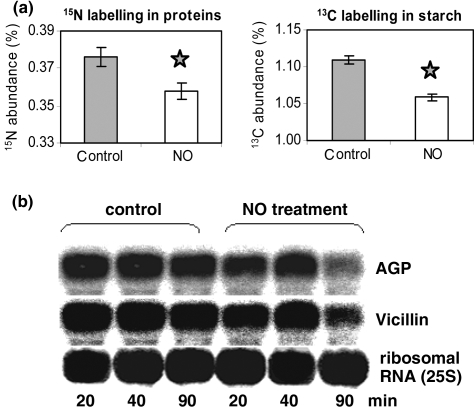
Effects of nitric oxide (NO) on storage metabolism. Pea (*Pisum sativum*) embryos were incubated *in vitro* with ^15^N-labelled glutamine and ^13^C-labelled sucrose. (a) Response of isotope abundance in the protein and starch fractions of pea embryos to the presence of 1.5 µm NO after 5 h of incubation. Labelling is given as the percentage of total carbon and total nitrogen, respectively. Data are shown as means ± SD. (b) Northern blot analysis of transcript accumulation for ADP-glucose pyrophosphorylase (AGP) and vicillin in *in vitro* incubated pea embryos at different time intervals in response to the presence of 1.5 µm NO. Stars indicate statistically significant differences according to *t*-test (*P* < 0.05).

It is known that the synthesis of storage products in seeds is an energy-limited process (Neuhaus & Emes2000), and that local ATP availability is correlated with a high localized level of biosynthetic activity (Borisjuk *et al*., 2003; Rolletschek *et al*., 2003, 2004, 2005a,b; Weber *et al*., 2005). Thus, the transient suppression of ATP synthesis may be a significant determinant of the level of biosynthetic activity in the seed and of its adjustment to a changing environment (particularly the transition from darkness to light and vice versa in photosynthetically active seeds). We therefore examined the effect of exogenously supplied NO on steady-state metabolite concentrations and fluxes in pea embryos using an *in vitro* system, where NO was delivered via microinjection to establish a stable concentration of 1.5 µm NO in the nutrient buffer. As revealed by targeted metabolite analysis, this treatment resulted in significantly increased concentrations both of lactate and of pyruvate, isocitrate and succinate (Table 2). The latter may well be a direct consequence of NO-inhibited respiratory fluxes in the citric acid cycle (Wendehenne *et al*., 2001), but the effect on lactate concentrations appears to reflect a shift from aerobic to anaerobic metabolism. In a second set of experiments, pea embryos were treated with NO as above and incubated in the presence of ^13^C-sucrose and ^15^N-glutamine. Subsequently, the uptake of isotopes and their partitioning into the various storage products were analysed. Exogenously supplied NO significantly decreased label incorporation into both starch and proteins (Fig. 4a,b), clearly indicating lower biosynthetic activity, and thereby supporting the conclusions drawn from steady-state metabolite concentrations.

**Table 2:** Primary metabolites and their response to nitric oxide (NO) treatment

	Control		+ NO	
		
	Mean	SE	Mean	SE
Soluble sugars				
Glucose	0.1	0.1	0.0	0.0
Fructose*	0.6	0.2	0.8	0.1
Sucrose*	39.1	5.6	24.9	2.2
Nucleotide sugars				
UDP-glucose†	0.01	0.00	0.01	0.00
Glycolytic intermediates				
Glucose-1-phosphate†	0.8	0.3	1.7	0.3
Glucose-6-phosphate†	9.0	3.1	3.3	0.4
Fructose-6-phosphate†	3.0	0.9	1.1	0.2
3-Phosphoglycerate†	0.8	0.3	0.7	0.2
Phosphoenolpyruvate†	0.07	0.02	0.07	0.01
Pyruvate†	2.3	0.5	**4.9**	1.0
Lactate†	1.5	0.4	**3.7**	0.6
Organic acids				
Citrate†	6.3	0.8	5.9	0.7
Isocitrate†	1.7	0.2	**3.0**	0.2
*cis*-Aconitate†	2.1	0.6	2.5	0.3
Fumarate†	0.3	0.0	0.4	0.0
Oxoglutarate†	6.6	1.0	10.1	1.4
Succinate†	1.7	0.3	**4.2**	0.2
Malate†	5.8	0.6	6.9	0.4

Bold values indicate significant differences according to a *t*-test (*P* < 0.05).

* Values are in µmol g^−1^ FW.

† Values are in relative units.

Because primary NO effects are immediate responses (Figs 1–3), adjustments to the energy and storage metabolism are presumably regulated post-transcriptionally. However, long-term NO treatment (1.5 µm) may also induce changes at the molecular level. The expression of two major storage-related genes (ADP-glucose pyrophosphorylase (starch biosynthesis) and vicillin (storage protein biosynthesis)) was not affected by the presence of NO in pea embryos cultured *in vitro* for at least 40 min, but was decreased thereafter (Fig. 4c,d).

We conclude that local ATP availability and the biosynthetic activity of seeds are regulated by NO, probably via its respiratory control function. Of course, this does not exclude the possibility that NO effects also involve other mechanisms such as protein nitrosylation (Lindermayr *et al*., 2005) and gene expression changes (Huang *et al*., 2002).

### Concluding remarks

**Fig. 5:**
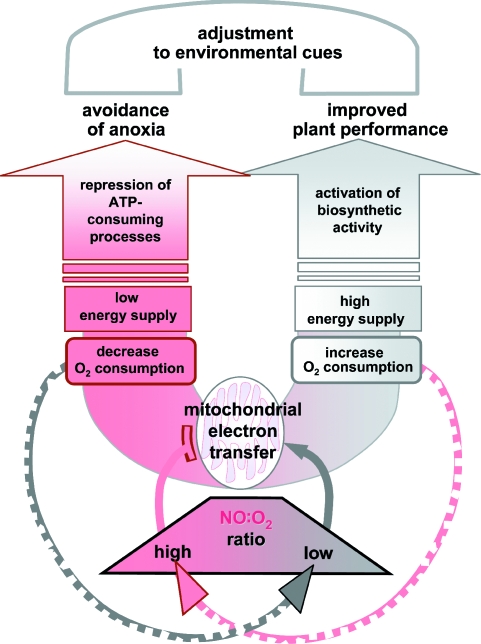
Model of the role of nitric oxide (NO) in low-oxygen sensing and balancing, and the control of energy and storage metabolism in seeds. Under O_2_ shortage, both the stability and the synthesis of NO increase, causing elevated NO:O_2_ ratios. NO inhibits mitochondrial electron transport via the cytochrome pathway. The efficiency of this inhibition depends on the endogenous O_2_ concentration. Thus, the NO:O_2_ ratio triggers oxygen balancing in an auto-regulatory manner, avoiding seed anoxia. At high NO:O_2_ ratios, mitochondrial inhibition conserves O_2_ and tends to increase O_2_ availability. Concomitantly, the decrease in ATP availability represses ATP-consuming processes, including storage activity. Increasing oxygen availability, for example via seed photosynthesis or environmental cues, decreases the NO:O_2_ ratio. This suspends mitochondrial inhibition, increases O_2_ consumption and ATP availability, and eventually promotes the biosynthetic activity of seeds. In conclusion, NO balances endogenous O_2_ concentrations and allows the seed to adjust metabolism to O_2_ availability, which may rapidly change depending on the natural environment.

Overall, the major indications are that NO mediates the integration of low-oxygen sensing, oxygen balancing, respiratory control, ATP availability and storage activity in seeds (Fig. 5). Oxygen availability controls the endogenous concentration of NO, and NO in turn regulates the rate of oxygen consumption, that is, oxygen availability. In addition, the inhibitory effect of NO on the mitochondrial cytochrome oxidase is dependent on oxygen concentration. This twofold feedback regulation may confer a fairly well-balanced steady-state oxygen concentration under O_2_-limited conditions. Because NO is sensitive to molecular oxygen (intrinsic chemical link), NO might be regarded as an oxygen sensor: it reacts directly with O_2_ and triggers a hypoxic response.

The NO-mediated control of metabolic activity is probably not restricted to seeds, but represents a universal mechanism for oxygen sensing and balancing in plants. Recent data obtained by Stoimenova *et al*. (2003) show that roots lacking nitrate reductase activity have higher fermentation rates as well as ATP concentrations. Libourel *et al*. (2006) demonstrated that nitrite rather than nitrate has beneficial effects on pH regulation and flooding tolerance. Such findings are very well explained by our model presented in Fig. 5: in the absence of nitrite, NO synthesis might be reduced. Mitochondrial respiration is less restricted, ATP concentrations rise, but endogenous O_2_ concentrations decrease, which in turn promotes fermentation activity. Vice versa, in the presence of nitrite endogenous oxygen is balanced (via NO) to concentrations avoiding/reducing cytoplasmic acidosis.

Key components of NO signalling pathways, identified in the mammalian system, have also been found in plants, and these fit well into our hypothesized framework: (1) both anoxia and NO treatment cause a transient increase in the concentration of cyclic GMP (Pfeiffer *et al*., 1994; Reggiani1997); (2) plant proteins with various cellular functions, including glycolytic enzymes, are reversibly S-nitrosylated upon NO treatment (Lindermayr *et al*., 2005); and (3) NO affects calcium fluxes, protein phosphorylation, alternative oxidase and aconitase (Wendehenne *et al*., 2001; Neill *et al*., 2003). There appears to be a strong similarity between the NO-mediated signalling mechanisms in plants and mammals. Investigations of the molecular mechanisms linking hypoxically induced NO to reductions in storage activity are required. This will provide a more comprehensive view of NO signalling in seeds, and offer a new perspective for biotechnological crop improvement.

In plants, endogenous NO is metabolized via nonsymbiotic haemoglobins (Perazzolli *et al*., 2004). Down-regulation of haemoglobin expression results in elevated NO concentrations and lower ATP concentrations upon NO treatment, even in the presence of 40% oxygen (Dordas *et al*., 2003). This compromised ability to use oxygen adequately has been labelled ‘metabolic hypoxia’ and is thought to occur during sepsis and other inflammatory/degenerative diseases in mammals (Moncada & Erusalimsky2002). Overexpression of haemoglobins promotes early plant growth under hypoxia (Hunt *et al*., 2002). Assuming that the embryo develops under permanent hypoxia, similar effects might take place in seeds overexpressing haemoglobins. We propose that the transgenic manipulation of NO metabolism in seeds might represent an efficient approach to promote energy availability for biosynthesis, and offer a promising perspective for future improvement of crop productivity.

## Supplementary material

The following supplementary material is available for this article online:

Fig. S1 The dynamics of endogenous oxygen concentration in rapeseed (*Brassica napus*) in response to light:dark transitions.

Fig. S2 Nitric oxide (NO) release by pea (Pisum sativum)embryos measured using the membrane inlet mass spectrometry (MIMS) technique (see the Materials and Methods).

Fig. S3 Effect of nitric oxide (NO) on oxygen uptake by pea (*Pisum sativum*) embryos measured using the membrane inlet mass spectrometry (MIMS) technique. This material is available as part of the online article from http://www.blackwell-synergy.com/doi/abs/10.1111/j.1469-8137.2007.02226.x (This link will take you to the article abstract.) Please note: Blackwell Publishing are not responsible for the content or functionality of any supplementary materials supplied by the authors. Any queries (other than about missing material) should be directed to the New Phytologist Central Office.

About New Phytologist

- New Phytologist is owned by a non-profit-making **charitable trust** dedicated to the promotion of plant science, facilitating projects from symposia to open access for our Tansley reviews. Complete information is available at www.newphytologist.org.
- Regular papers, Letters, Research reviews, Rapid reports and both Modelling/Theory and Methods papers are encouraged. We are committed to rapid processing, from online submission through to publication ‘as-ready’ via *OnlineEarly* – our average submission to decision time is just 30 days. Online-only colour is **free**, and essential print colour costs will be met if necessary. We also provide 25 offprints as well as a PDF for each article.
- For online summaries and ToC alerts, go to the website and click on ‘Journal online’. You can take out a **personal subscription** to the journal for a fraction of the institutional price. Rates start at £135 in Europe/$251 in the USA & Canada for the online edition (click on ‘Subscribe’ at the website).
- If you have any questions, do get in touch with Central Office (newphytol@lancaster.ac.uk; tel +44 1524 594691) or, for a local contact in North America, the US Office (newphytol@ornl.gov; tel +1 865 576 5261).
