# Supplementary figures and images for: Low oxygen sensing and balancing in plant seeds: a role for nitric oxide
Source: New Phytol. 2007 Dec;176(4):813–23. doi: 10.1111/j.1469-8137.2007.02226.x (PMC2440544; doi:10.1111/j.1469-8137.2007.02226.x)

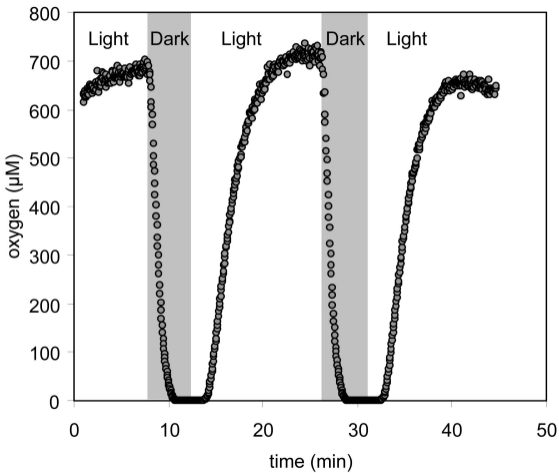

Supplement: Fig. S1 — The dynamics of endogenous oxygen concentration in rapeseed (Brassica napus) in response to light:dark transitions. [file nph0176-0813-SD1.pdf]

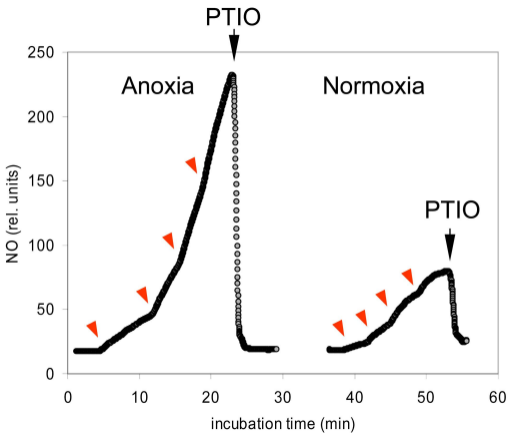

Supplement: Fig. S2 — Nitric oxide (NO) release by pea (Pisum sativum)embryos measured using the membrane inlet mass spectrometry (MIMS) technique (see the Materials and Methods). [file nph0176-0813-SD2.pdf]

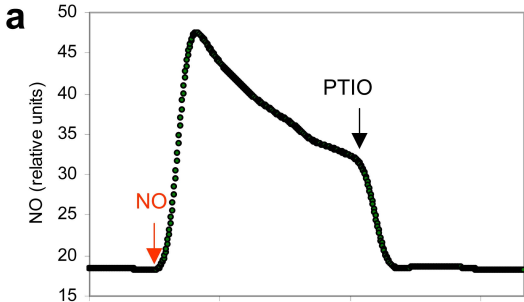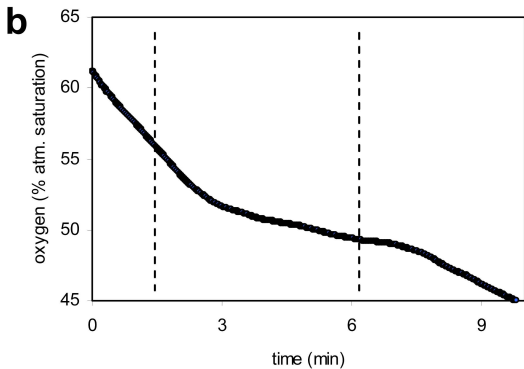

Supplement: Fig. S3 — Effect of nitric oxide (NO) on oxygen uptake by pea (Pisum sativum) embryos measured using the membrane inlet mass spectrometry (MIMS) technique. [file nph0176-0813-SD3.pdf]
